# Mutual Solubility Study in Supercritical Fluid Extraction of Tocopherols from Crude Palm Oil Using CO2 Solvent

**DOI:** 10.3390/ijms11103649

**Published:** 2010-09-28

**Authors:** Reza Davarnejad, Zainal Ahmad, Suhairi A. Sata, Mostafa Keshavarz Moraveji, Farzaneh Ahmadloo

**Affiliations:** 1 Chemical Engineering Department, Arak University, Arak, Iran; E-Mails: m-moraveji@araku.ac.ir (M.K.M.); fa.ahmadlo@yahoo.com (F.A.); 2 School of Chemical Engineering, Engineering Campus, Universiti Sains Malaysia, 14300 Nibong Tebal, Penang, Malaysia; E-Mails: chzahmad@eng.usm.my (Z.A.); chhairi@eng.usm.my (S.A.S.)

**Keywords:** crude palm oil, phase behavior, supercritical extraction, tocopherols

## Abstract

In this article, the mutual solubility of tocopherols from crude palm oil was studied using carbon dioxide as a solvent at the temperatures of 80, 100 and 120 °C. Each sample from the phase equilibrium unit contained two parts. The liquid part was analyzed by gas chromatography (GC) in order to measure the tocopherol composition and, on the other hand, the vapor phase was conducted in an expansion vessel in order to measure the pressure increment during the expansion process. Two phase equilibrium data was calculated using the liquid phase composition and pressure increments during the expansion process. Results showed that the maximum solubility of tocopherols was around 2.27% at a temperature of 120 °C and at pressure of 5.44 MPa.

## 1. Introduction

The main product from palm fruits usually is palm oil. In order to get palm oil, the palm fruits are first treated with steam and, thereafter, the oil-containing parts are separated from the bunch. By mechanical extraction of these parts, crude palm oil (CPO) is obtained. CPO is a semi solid material at room temperature and has a melting point of 36 °C [1]. Therefore, to retain certain components in CPO, such as vitamin E (tocopherols), it is important to use an appropriate extraction process like supercritical extraction using CO_2_.

Extraction with carbon dioxide under supercritical conditions constitutes an emerging technology in terms of environmental impact. The advantages of using carbon dioxide include its lack of toxicity, chemical inertness, low cost and ready availability [2]. Furthermore, the use of carbon dioxide is also beneficial in terms of the final quality to the products obtained since this technique does not give rise to excessive heating, which usually has a negative effect on thermolabile compounds [3].

CPO contains several tocochromanols at a concentration of approximately 500 ppm. The investigated soy oil deodorized distillated contains 50 wt. % tocochromanols [4]. A total of eight substances collectively known as tocochromanols can be subdivided into two groups: the tocopherols and the tocotriennols with -trienols processing three unsaturated double bonds in their aliphatic side chain at the 3′, 7′ and 11′ position [4].

**Figure 1:**
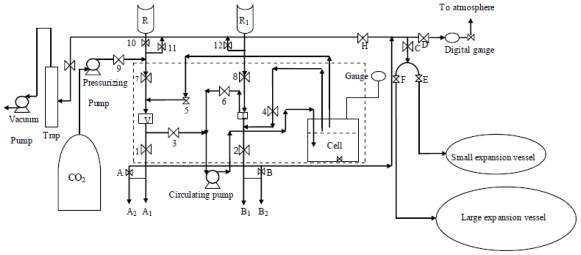
Phase equilibrium supercritical fluid extraction apparatus.

Each group consists of an α-, β-, γ- and δ-tocopherol (-trienol), referring to the number and site of methyl groups at the chroman-ring (R_1_ and R_2_ in Figure 1). Of these compounds, α-tocopherol is reported to have the highest biological activity [5]. The Vitamin E activity has been associated with the antioxidant properties of these components, especially against lipid peroxidation in biological membranes.

As for the previous studies applying supercritical fluid extraction, an overview of this process is given in [6]. Markom *et al.* described the supercritical CO_2_ fractionation of crude palm oil [7] and Güçlü-Üstündağ correlated the solubility behavior of minor lipid components in supercritical carbon dioxide [8]. Both studies showed that solubility differences between α-tocopherol and β-carotene were clearly obtained. On top of that, many authors have described the enrichment of Vitamin E from oil or oil deodorizer distillate with supercritical fluid extraction [4,9–14].

In this article, solubilities of tocopherols from CPO at temperatures of 80, 100 and 120 °C and at near critical pressure of solvent were studied and compared.

## 2. Experimental

### 2.1. Materials

Crude palm oil and CO_2_ were purchased from United Oil Palm Industries, Nibong Tebal, Malaysia and Mox Sdn. Bhd (99.99%), respectively. α-, β-, γ- and δ-tocopherol (95%) from Merck were used as standards, and acetone (99.99%) from Fisher Scientific and hexane (99.8%) from J. T. Baker were used for analysis.

### 2.2. Apparatus Set Up

The equipment used in this research was a phase equilibrium loading recirculation high-pressure type apparatus as shown in Figure 1. This equipment is suitable for determining equilibrium data as reported in [15]. The method, which involves the recirculation of the gas phase (using a circulating pump) through liquid, is widely used in determining the equilibrium data. This is because a vapor sample can be easily isolated by using a by-pass in the circulation loop. A substantial advantage of this method is that it is not necessary to inject any displacement fluid, such as mercury, into the equilibrium cell (extractor) in order to maintain a constant pressure during the extraction of the samples.

Furthermore, a short time is required for attaining equilibrium condition. In some of the equilibrating units which are similar to the one used in this study, a liquid sample is obtained by allowing the liquid to fall under gravity into a liquid sample bomb after equilibrium. In the apparatus, the attainment of equilibrium was further assisted by a magnetic stirrer which was installed in the equilibrium vessel [15].

All the units of the apparatus, *i.e.*, the equilibrium cell (volume 500 cm^3^), the joining tubes, the vapor and liquid sample bombs (for vapor and liquid equilibrium phases isolation, with volumes of 50 cm^3^ and 40 cm^3^ respectively), the couplings and the valves were made of stainless steel and were designed to withstand an operating pressure of up to 500 bar and an operating temperature of up to 200 °C. The equilibrium section of this apparatus is shown within the dashed line. The temperature in the equilibrium cell was measured by using a digital indicator temperature which was connected to a temperature sensor inside the cell. A pressure gauge was also connected to the cell in order to show the pressure inside the cell. In each sample which involved the liquid and gas phases, the liquid phase was collected in the sample-tube and the gas phase was expanded in a flash drum at the same time. Then the flash drum pressure was checked using a digital gauge which showed very low pressures. The small expansion vessel (for expansion of vapor phase of liquid sample obtained from the liquid sample bomb, volume: 7 liters) and the large expansion vessel (for expansion of vapor phase of vapor sample obtained from the vapor sample bomb, volume: 35 liters) was used when the liquid sample bomb and the vapor sample bomb were obtained.

The data reliability and uncertainty due to the measuring parts of this apparatus have been tested and are discussed in detail in the literature [16,17].

### 2.3. Experimental Procedure

Firstly, valves 3, 4, 5, 6, 7, 11 and 13 were opened while the rest were closed. The vacuum pump was switched on to create a vacuum in the cell. The vacuum pump was switched off and valves 11 and 13 were also closed after a few minutes. When the cell and the circuit were under vacuum (vacuum pressure around −0.5 psig), the feed under study (CPO, which was pre-heated up to 40 °C) was fed into the equilibrium cell. This was accomplished by first filling the reservoir (R_1_) with about 120 cm^3^ of feed. The liquid heavy component was then allowed to go directly into the cell by turning on valve 8 and turning off valve 6. Having charged the cell with the heavy component, CO_2_ at cylinder pressure was then admitted to the cell by using a high pressure pump which was connected between the cylinder and the cell.

Then, valve 9 was opened and immediately the high pressure pump was switched on. After a few minutes, valve 9 was closed and the pressurizing pump was switched off after the system had been pressurized.

In the filling process, the heaters for the cell, the air bath and also the bath fan were switched on. Valves 6 and 3 were turned off and the cell was carefully brought to the desired temperature. This was accomplished by controlling the heater outputs, which were gradually increased to obtain the required temperature. Equilibrium and sampling were achieved when the required temperature (by controlling the temperature indicator) was reached and had remained constant (equilibrium condition); the pneumatic re-circulating pump and the magnetic stirrer were then turned on at the same time.

During sampling, valve 6 was opened and after a few seconds valve 3 was also opened. The reason for this was to avoid any droplets of solvent from passing through the vapor sample bomb flow section. The pump and stirrer were left on for about 30 minutes in order to ensure that equilibrium had been achieved. When equilibrium was resumed, the pump and the stirrer were turned off and internal valves 3, 5, 4 and 6 were kept opened. The phases were then allowed to stand in contact with each other for about 30 minutes in order to allow any bubbles in the liquid to become disengaged. After 30 minutes, the vapor sample bomb was isolated by turning off valves 3 and 5 and the liquid sample bomb was isolated by turning off valves 4 and 6. Samples of the gas and liquid phases were then extracted from the vapor bomb and the liquid bomb through valves A and B (Figure 1).

### 2.4. Samples Analysis

The taken samples were then analyzed for the tocopherols. For this purpose, the following analytical procedure was carried out using a GC (brand: Perkin Elmer, US; model: Clarus 500) which was equipped with a column (Heliflex AT™ −35, 25 m × 0.25 mm, 0.2 μm), oven temperature was set to 310 °C, carrier gas was hydrogen with a velocity of 40 cm/sec and the employed detector was the FID to detect tocopherols.

The standard solutions of α-, β-, γ- and δ-tocopherol with the concentrations of 10, 25 and 50 ppm were diluted into a solvent mixture of 50 percent acetone and 50 percent hexane (v/v), separately prepared. These solutions, prepared by diluting these chemicals, were initially prepared with concentration of 500 ppm.

The GC diagram obtained from each sample (see Appendix A) was compared with the diagrams obtained from the standard materials. Then, by applying the standard equation [Samples concentration (ppm) = sample area/standard area × standard concentration], samples concentration in terms of each substance was calculated.

## 3. Results and Discussion

The system carbon dioxide-heavy components (tocopherols) were studied at temperatures of 80, 100 and 120 °C and at various pressures. High temperatures were chosen in this research because, according to the literature, high temperature had a good effect on the solubility of triacylglycerols in CO_2_ which also assisted the other CPO compounds (such as β-carotene and tocopherols) solubilities as an appropriate co-solvent in CO_2_ [1,7,16,17]. The compositions of the equilibrium phases were noted at each pressure. The mole fractions of heavy component in the liquid (x_1_) and vapor (y_1_) phases on the carbon dioxide-free basis were calculated from the following equations [1,15–17]:

(1) $$x_{1}=\frac{{\left({nx}_{1}\right)}^{L}}{\left[{\left({nx}_{1}\right)}^{L}+{\left({nx}_{2}\right)}^{L}\right]}$$

(2) $$y_{1}=\frac{{\left({nx}_{1}\right)}^{G}}{\left[{\left({nx}_{1}\right)}^{G}+{\left({nx}_{2}\right)}^{G}\right]}$$

where,

(nx_1_)^L^: number of moles of tocopherol in the liquid phase on a carbon dioxide free basis.

(nx_1_)^G^: number of moles of tocopherol in the vapor phase on a carbon dioxide free basis.

(nx_2_)^L^: number of moles of palm oil in the liquid phase on a carbon dioxide free basis.

(nx_2_)^G^: number of moles of palm oil in the vapor phase on a carbon dioxide free basis.

The calculations for the mole fraction of carbon dioxide in the liquid phase (x) and in the vapor phase (y) of this system were carried out using the Equations (3) and (4) [1,15–17].

(3) $$y=\frac{{\left(ny\right)}^{G}}{\left[{\left(nx\right)}^{G}+{\left(ny\right)}^{G}\right]}$$

(4) $$(1-x)=\frac{{\left(nx\right)}^{L}}{\left[{\left(ny\right)}^{L}+{\left(nx\right)}^{L}\right]}$$

where,

(ny)^G^: number of moles of carbon dioxide in the vapor sample bomb.

(nx)^G^: number of moles of heavy component in the vapor sample bomb.

(ny)^L^: number of moles of carbon dioxide in the liquid sample bomb.

(nx)^L^: number of moles of heavy component in the liquid sample bomb.

The values of (nx)^L^ and (nx)^G^ were calculated from the following relationship [1]:

(5) $${\left(nx\right)}^{L}\text{or}{\left(nx\right)}^{G}=\frac{\left(\text{weight of extracted heavy components}\right)}{\left[x_{A}M_{A}+(1-x_{A})M_{B}\right]}$$

where,

x_A_ = mole fraction of heavy component in the liquid sample bomb (or in the vapor sample bomb).

x_B_ = mole fraction of the other substances (except heavy component) in the liquid sample bomb (or in the vapor sample bomb).

M_A_ = molecular weight of heavy component.

M_B_ = average molecular weight of the other substances.

In order to calculate (ny)^G^ and (ny)^L^, it is necessary to consider the deviation from the perfect gas law pressure of about 1 bar where it is expressed by the following equation [1,15–18]:

(6) $$PV=n(RT+B_{v}P)$$

P: pressure, V: system volume, n: number of moles of gas, B_v_: second virial coefficient. This equation leads directly to the following expression [1,15–17]:

(7) $${\left(ny\right)}^{G}=V\left[\frac{P_{2}}{RT+B_{v}P_{2}}-\frac{P_{1}}{RT+B_{v}P_{1}}\right]={VP}_{2}\left[\frac{1-(\frac{P_{1}}{P_{2}})(\frac{RT+B_{v}P_{2}}{RT+B_{v}P_{1}})}{\left(RT+B_{v}P_{2}\right)}\right]=\frac{V\times \Delta P}{RT+B_{v}\times \Delta P}$$

for the number of moles of gas in the vapor sample bomb where, P_1_ and P_2_ are expansion vessel pressure before and after expansion, ΔP is pressure increment before and after the expansion process and V is the volume of the system (35 liters). The second virial coefficient was obtained for carbon dioxide (solvent) from literature [18].

The number of moles of CO_2_ in the liquid sample bomb, (ny)^L^, is calculated by applying the same procedure as that which is given above except that the total volume of the expansion system (V) is taken as 7 liters.

The mole fractions of CO_2_ in equilibrium supercritical fluid extraction of tocopherols (α-, β-, γ-, δ-tocopherol) for the liquid and vapor phases at temperatures of 80, 100, 120 °C are shown in Table 1. It is clearly shown that the mole fractions of tocopherols in the vapor phase increased with increasing the pressure at a temperature of 80 °C. At low temperatures, it is shown that it does not have a considerable effect on tocopherols separation from the liquid phase, while in high pressures it can properly affect on tocopherols separation. These phenomena were also observed and supported by literature [19]. Although a regular trend was not observed in vapor phases at 100 and 120 °C, a maximum solubility of the tocopherols was observed at 7.68 MPa and at 100 °C as well as at 5.44 MPa and at 120 °C, respectively. The pressure and temperature affected the tocopherol solubility in CO_2_ although the temperature effect in CPO was more significant. It seems that the irregular trend which is observed for triacylglycerols (TAGs) [1,17] to legitimize this irregularity for tocopherols because TAGs can assist other substances solubilities (from CPO) in CO_2_ as a co-solvent [1,7,16,17]. The positive effect of pressure on the solubility of tocopherols has been reported and supported in the literature [4]. The optimum conditions around these operating regions for the solubility of tocopherols were observed at 120 °C and at 5.44 MPa.

**Figure 2:**
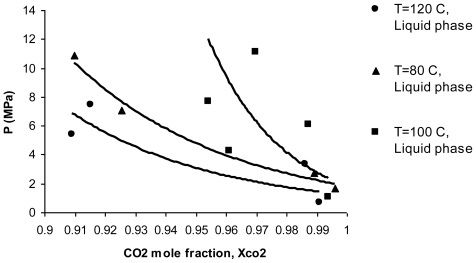
Liquid phase equilibrium diagram for α-tocopherol at 80, 100 and 120 °C.

**Figure 3:**
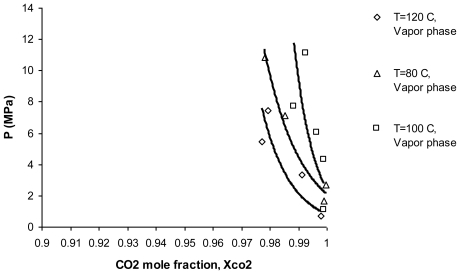
Vapor phase equilibrium diagram for α-tocopherol at 80, 100 and 120 °C.

Liquid and vapor phase diagrams for α-tocopherol substance at temperatures of 80, 100 and 120 °C are shown in Figures 2 and 3, respectively (these graphs can be also expanded to all of the tocopherols because the differences between each type of tocopherol at a constant temperature and pressure is very low). According to Figure 2, high temperature increases α-tocopherol solubility in the liquid phase, increasing the pressure increases the solubility as well. This demonstrates that high solubility of tocopherols *versus* temperature in the liquid phase promotes higher solubility of tocopherols in the vapor phase, as shown in Figure 3.

In Figure 3, it is clearly shown that high temperature increases α-tocopherol solubility in the vapor phase as well as by increasing the pressure [19].

Equilibrium ratio factors (K_i_ = y_i_/x_i_) are shown in Table 2. The tocopherols were enriched in the liquid phase because the equilibrium ratio is always less than 1. This result is in good agreement with the results obtained by Gast *et al.* [4]. As shown in Table 2, temperature increased the solubility of tocopherols in the vapor phase because (K_tocopherols_)_120 °C_ > (K_tocopherols_)_100 °C_ > (K_tocopherols_)_80 °C_. As seen in this table, the K ratio for all pressures was around 0.2 while 0.6 at a pressure of 3.33 MPa. According to the experimental work published by Gast *et al.* [4], the K ratio showed a constant trend (around 0.2) at high pressures and it sharply increased at low pressures, although they only studied a mild temperature of 67 °C.

Statistical analysis software (SPSS software, version 17) was applied to analyze the obtained data statistically and optimize the operating conditions. For the analysis, One-Sample Kolmogorov-Smirnov Test was applied for each column of Table 1 with the pre-assumption of normal data. Since the *P value* was always more than 0.5, our initial assumption was approved. Then, the Pearson Correlation coefficient for data correlation was used. According to the Correlation Matrix, it was observed that when one component in the vapor phase (main phase which shows the solubility) increased, the other components increased. Furthermore, all data in the vapor phase had a high negative correlation with pressure, meaning that solubility often increases with increasing pressure. In the text stage, One Way Anova for each component at each temperature was used to analyze the Means Equality Hypothesis. Since data groups mean were very near to each other therefore, all tests were not applicable in level of 0.05. According to the Means Difference and Standard Deviation, at a temperature of 120 °C and pressure of 5.44 MPa, the data had the lowest Means Difference (97.73) and Standard Deviation (0.00). The Mean plot *versus* temperature for α-tocopherol (as an example) is shown in Appendix B.

## 4. Conclusions

The maximum solubility of tocopherols (based on the vapor phase) was around 2.27% at 5.44 MPa and at 120 °C. This result means that an intermediate pressure and at a rather high temperature were the most suitable conditions for the solubility of tocopherols in CO_2_. Although the composition of the tocopherols from CPO slightly changed, from the scale up point of view the reported data would be applicable. This could be due to the pressure increment that considerably influenced the calculated data. On the other hand, high solubility of TAGs in CO_2_ may assist as a co-solvent in high solubility of tocopherols. Furthermore, supercritical fluid extraction process using CO_2_ was an effective crude palm oil enrichment technique from tocopherols (vitamin E) as the K ratio always was less than one.

## Figures and Tables

**Appendix A:**
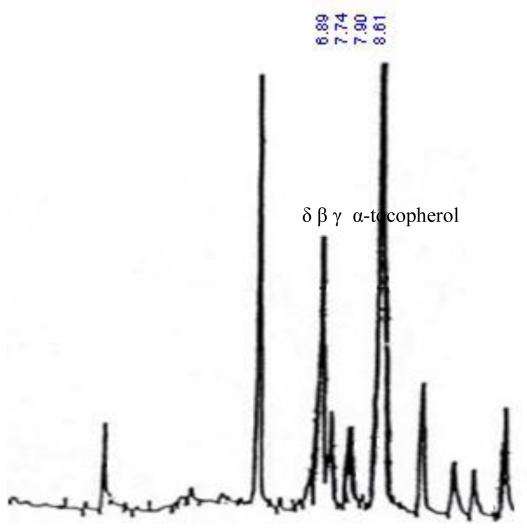
Gas chromatogram for Tocopherols.

**Appendix B:**
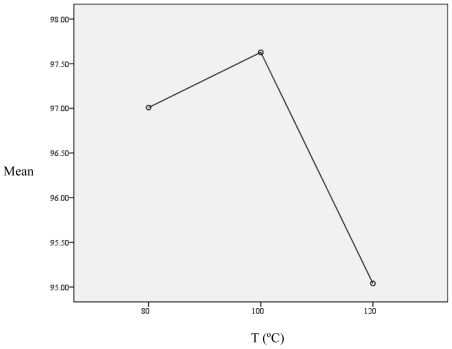
The Mean plot *versus* temperature for α-tocopherol obtained from the SPSS.

**Table 1:** CO_2_ mole percentages in equilibrium supercritical extraction of tocopherols for liquid and vapor phases at temperatures of 80, 100 and 120 °C.

P (MPa) T = 80 °C	Liquid phase, CO_2_ mole percentage related to α-tocopherol	Vapor phase, CO_2_ mole percentage related to α-tocopherol	Liquid phase, CO_2_ mole percentage related to β-tocopherol	Vapor phase, CO_2_ mole percentage related to β-tocopherol	Liquid phase, CO_2_ mole percentage related to γ-tocopherol	Vapor phase, CO_2_ mole percentage related to γ-tocopherol	Liquid phase, CO_2_ mole percentage related to δ-tocopherol	Vapor phase, CO_2_ mole percentage related to δ-tocopherol
10.88	91.00	97.81	91.00	97.81	91.00	97.81	91.00	97.81
7.10	92.54	98.51	92.54	98.52	92.54	98.52	92.54	98.52
2.72	98.89	99.97	95.58	98.89	95.58	98.89	95.58	98.89
1.70	99.61	99.90	99.61	99.90	99.61	99.90	99.61	99.90

T = 100 °C								
11.08	96.98	99.26	96.98	99.26	96.98	99.26	96.98	99.26
7.68	95.42	98.86	95.42	98.86	95.42	98.02	95.42	98.86
6.05	98.73	99.65	98.73	99.65	98.73	99.65	98.73	99.65
1.08	99.38	99.91	99.41	99.91	99.41	99.91	99.41	99.91

T = 120 °C								
7.48	91.53	97.92	91.53	97.92	91.53	97.92	91.53	97.92
5.44	90.92	97.73	90.92	97.73	90.92	97.73	90.92	97.73
3.33	98.61	99.12	98.61	99.12	98.61	99.12	98.61	99.12
0.68	99.10	99.78	99.10	99.78	99.10	99.78	99.10	99.78

**Table 2:** Equilibrium ratio factors of tocopherols *versus* pressure at 80, 100 and 120 °C.

P (MPa) T = 80 °C	10.88	7.10	2.72	1.70

K_α-tocopherol_	0.243	0.199	0.230	0.254
K_β-tocopherol_	0.243	0.198	0.250	0.254
K_γ-tocopherol_	0.243	0.198	0.250	0.254
K_δ-tocopherol_	0.243	0.198	0.250	0.254

P (MPa) T = 100 °C	11.08	7.68	6.05	1.08

K_α-tocopherol_	0.244	0.249	0.274	0.147
K_β-tocopherol_	0.244	0.249	0.274	0.156
K_γ-tocopherol_	0.244	0.432	0.274	0.156
K_δ-tocopherol_	0.244	0.249	0.274	0.156

P (MPa) T = 120 °C	7.48	5.44	3.33	0.68

K_α-tocopherol_	0.245	0.250	0.634	0.246
K_β-tocopherol_	0.245	0.250	0.634	0.246
K_γ-tocopherol_	0.245	0.250	0.634	0.246
K_δ-tocopherol_	0.245	0.250	0.634	0.246
